# Characteristics of Gait Ataxia in δ2 Glutamate Receptor Mutant Mice, *ho15J*

**DOI:** 10.1371/journal.pone.0047553

**Published:** 2012-10-15

**Authors:** Eri Takeuchi, Yamato Sato, Eriko Miura, Hiroshi Yamaura, Michisuke Yuzaki, Dai Yanagihara

**Affiliations:** 1 Graduate School of Arts and Sciences, The University of Tokyo, Komaba, Meguro-ku, Tokyo, Japan; 2 Department of Physiology, School of Medicine, Keio University, Shinjuku-ku, Tokyo, Japan; 3 Core Research for Evolutional Science and Technology, Japan Science and Technology Corporation, Chiyoda-ku, Tokyo, Japan; Tokyo Metropolitan Institute of Medical Science, Japan

## Abstract

The cerebellum plays a fundamental, but as yet poorly understood, role in the control of locomotion. Recently, mice with gene mutations or knockouts have been used to investigate various aspects of cerebellar function with regard to locomotion. Although many of the mutant mice exhibit severe gait ataxia, kinematic analyses of limb movements have been performed in only a few cases. Here, we investigated locomotion in *ho15J* mice that have a mutation of the δ2 glutamate receptor. The cerebellum of *ho15J* mice shows a severe reduction in the number of parallel fiber-Purkinje synapses compared with wild-type mice. Analysis of hindlimb kinematics during treadmill locomotion showed abnormal hindlimb movements characterized by excessive toe elevation during the swing phase, and by severe hyperflexion of the ankles in *ho15J* mice. The great trochanter heights in *ho15J* mice were lower than in wild-type mice throughout the step cycle. However, there were no significant differences in various temporal parameters between *ho15J* and wild-type mice. We suggest that dysfunction of the cerebellar neuronal circuits underlies the observed characteristic kinematic abnormality of hindlimb movements during locomotion of *ho15J* mice.

## Introduction

The cerebellum has a long-recognized but poorly understood role in the control of locomotion. Most of the evidence for this role comes from patients with cerebellar damage who display balance abnormalities and gait ataxia characterized by abnormal coordination of limb movements [1], [2]. The cerebellar patients show incoordinated movements of the limb joints that particularly affect the ankles and knees [3]. Gait ataxia has been also investigated in animal studies, particularly through use of mice with mutation or knockout of genes and proteins of interest. For example, cerebellar gait ataxia has been examined in mice with mutation of metabotropic glutamate receptor-subtype 1, γ isoform of protein kinase C, phospholipase C β4, and δ2 glutamate receptor (GluD2) [4], [5], [6], [7], [8]. GluD2 is predominantly expressed in the distal dendrites of the Purkinje cells (PCs). Mice that are null for GluD2 have a reduced number of parallel fiber (PF)-PC synapses, multiple climbing fiber (CF) innervations, and deficit of long-term depression (LTD) [6], [9]. The importance of GluD2 in adaptive control of locomotion is also shown by the effect of injection of a function-blocking anti-GluD2 antibody into the subarachnoid space of the cerebellum; this treatment impaired the performance of wild-type mice in a rotarod test [10]. The mouse *hotfoot* mutation, *ho15J*, has been shown to be the result of an intragenic deletion of a gene encoding GluD2. The mutated GluD2 protein is retained in the endoplasmic reticulum, degraded, and is not transported to the dendritic spines of the PCs. The *ho15J* mice have a deficit of LTD and impaired performance on footprint and rotarod tests [11]. The ‘footprint’ and ‘rotarod’ performance tests are the most commonly used methods for assessing gait ataxia. Although the rotarod test is a relatively well-validated measurement, there are factors that need to be taken into account when assessing the results: body weights and fatigue may affect performance; also, some animals may refuse to participate [12].

Similarly to other GluD2 mutant mice, *ho15J* mice are not able to walk stably on a rotating rod [6], [11], [13]; therefore, we hypothesized that *ho15J* mice have severe incoordination of the limb movements, which is often seen in cerebellar patients [1], [2]. Moreover, we do not know whether *ho15J* mice are appropriate as a model of ataxic gait in cerebellar patients. In this study, we analyzed hindlimb kinematics during treadmill locomotion because most previous kinematic analyses during walking on a treadmill and/or flat runway in rodent models have been carried out on hindlimb behavior [14], [15], [16], [17], [18]. Moreover, use of a treadmill enables investigation of adaptability to altered belt speeds [5], [19].

**Figure 1:**
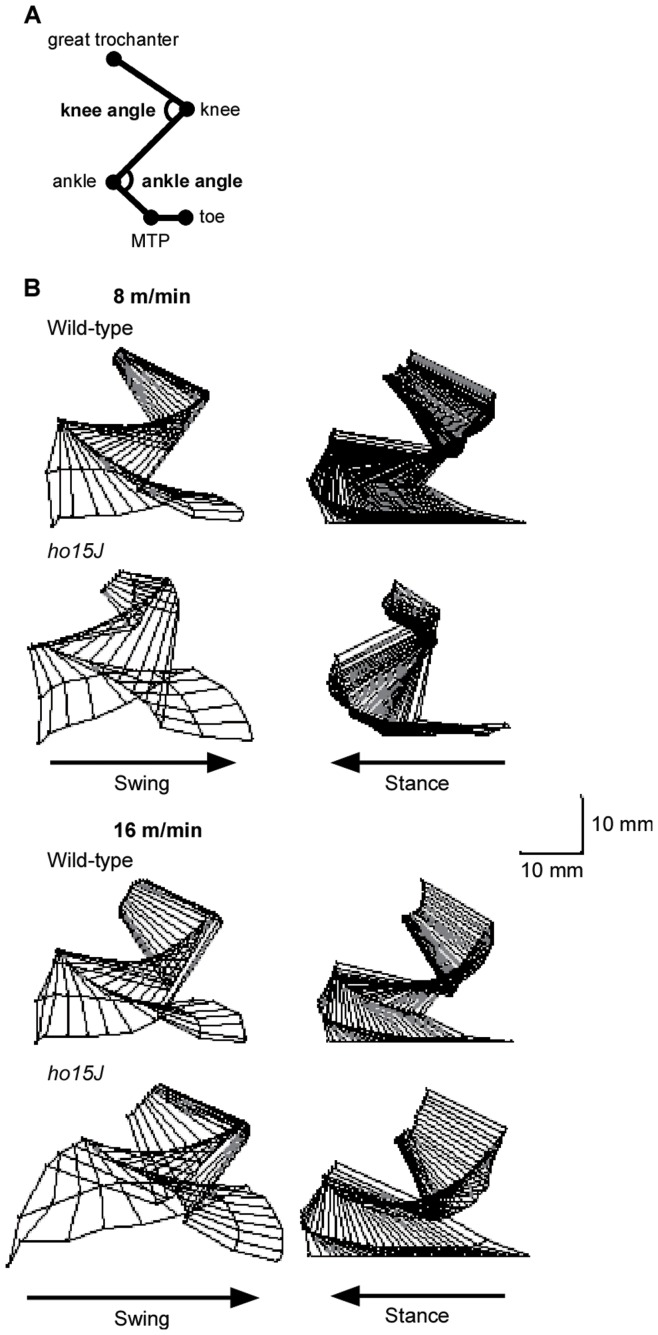
Stick figures of the right hindlimb during the step cycle. A: Positions of the reflective markers. Reflective markers were placed on the following anatomical landmarks: great trochanter, knee, ankle, 5th metatarsophalangeal joint (MTP), and a toe. B: Typical examples of stick figures of a step cycle (swing and stance phases) at different treadmill speeds for a *ho15J* mouse and a wild-type mouse. Scale bar indicates 10 mm.

## Materials and Methods

### Animals

This study was approved by the Ethical Committee for Animal Experiments at the University of Tokyo, and was carried out in accordance with the Guidelines for Research with Experimental Animals of the University of Tokyo and the Guide for the Care and Use of Laboratory Animals (NIH Guide, revised in 1996). Mice carrying the *ho15J* mutation (C3H background) were purchased from the Jackson Laboratory (Bar Harbor, ME, USA). Genotyping was performed using PCR as described previously [11]. Experiments were performed using *ho15J* homozygotes (n = 8, male, 8–12 weeks old) and wild-type C3H mice (n = 8, male, 8 weeks old) as controls. Wild-type C3H mice were supplied by CLEA (Tokyo, Japan). The animals were kept in a temperature-controlled room with a regular light/dark cycle (lights on from 08∶00 to 20∶00), and had ad lib access to food and water. The well-being of the mice was carefully monitored, and all efforts were made to minimize the number of animals used and any suffering in the course of the experiments.

**Table 1:** Average step cycle duration at 8 m/min.

			Mice		Statistics	
			Wild-type	*ho15J*		
			(Mean ± SEM)	(Mean ± SEM)	*t*	*p*
**Step cycle duration (ms)**			322.7±12.7	309.9±20.5	t(14) = 0.530	0.604
**Swing phase duration (ms)**			67.5±2.5	74.8±4.2	t(14) = 1.478	0.162
**Stance phase duration (ms)**			245.2±12.9	225.4±18.2	t(14) = 0.884	0.392

**Table 2:** Average step cycle duration at 16 m/min.

			Mice		Statistics	
			Wild-type	*ho15J*		
			(Mean ± SEM)	(Mean ± SEM)	*t*	*p*
**Step cycle duration (ms)**			221.9±4.0	218.3±9.7	t(9.371) = 0.343♦	0.739
**Swing phase duration (ms)**			68.9±1.7	67.4±5.1	t(14) = 0.291	0.775
**Stance phase duration (ms)**			143.0±2.9	141.4±5.2	t(14) = 0.267	0.793

♦ The statistical analysis was carried out using Welch’s test.

### Locomotion Recording

Before recording locomotion patterns, the mice were habituated to the treadmill apparatus and trained to walk on it. To enable observation of hindlimb movements, the fur on the hindlimb of each animal was shaved under isoflurane gas anesthesia (3% for induction, 1–2% for maintenance). Circular reflective markers (2.5 mm diameters) were precisely placed on the shaved skin of the right hindlimb at the great trochanter (hip), the knee, the lateral malleolus (ankle), the fifth metatarsophalangeal joint (MTP), and a toe. The mice were allowed to recover completely from the anesthesia before being placed on the treadmill. The animals walked freely at different speeds (8, 16, and 24 m/min) imposed by the treadmill belt and their locomotory movements were recorded at 200 frames per second using a high-speed digital image camera system (HAS-200, DITECT, Inc., Tokyo, Japan). The captured images were stored electronically for later analysis.

**Figure 2:**
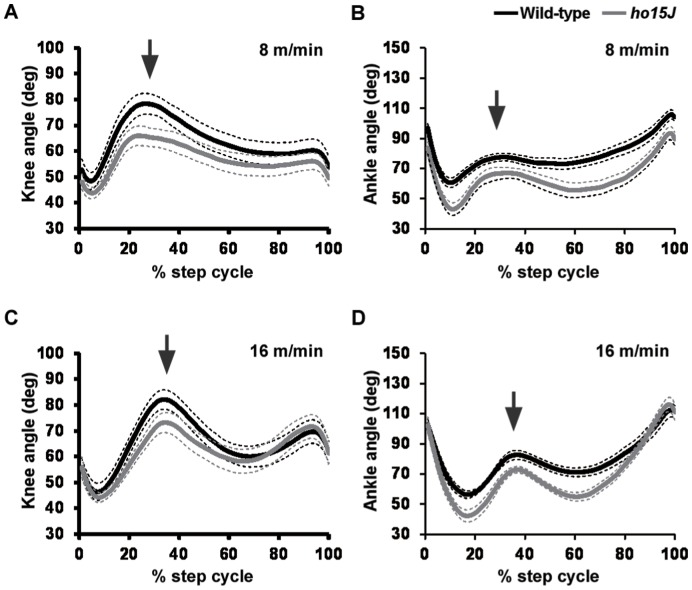
Joint angle displacement during the step cycle. Average knee and ankle angular displacements during step cycles at different treadmill speeds. Knee angle and ankle angle of the step cycle were normalized to obtain 100 samples per step cycle. Arrows indicate the phase change point (swing to stance). Dotted lines indicate SEM. A, C: knee angles; B, D: ankle angles.

### Motion Analysis

Motion analysis was limited to the sagittal plane parallel to the direction of walking. Custom-designed image analysis software (DIPP-Motion 2D, DITECT, Inc., Tokyo, Japan) was used to extract the two-dimensional coordinates of the various joint markers and to reconstruct a stick diagram representation of the right hindlimb. Due to skin slippage above the knee joint during walking, the actual knee position was corrected by triangulation from the position of the hip and ankle joint, using the measured lengths of the femur and tibia. In this study, we defined a step cycle as always having a phase in which the mouse supported its body weight with both hindlimbs (bisupport phase). If there was no bisupport phase in the observed cycle, then we excluded the data from analysis because the mouse was unable to walk. Temporal parameters were examined for 20 step cycles; 10 step cycles were used for kinematic analysis of each mouse. The step cycle can be divided into the swing phase and the stance phase. The swing phase is defined as starting at the moment when the animal lifts its foot from the treadmill belt and as ending when the foot comes back into contact with the treadmill belt. The stance phase is defined as starting when the foot touches the treadmill belt and as ending when the foot lifts from the belt. To analyze the angular excursions of the knee and ankle during a cycle, the step cycle duration was normalized, and cubic-spline interpolation was applied to the original data on the joint angles of the knee and ankle to obtain 100 samples per step cycle regardless of their duration using MATLAB computer software (MathWorks Inc., Natick, MA).

**Figure 3:**
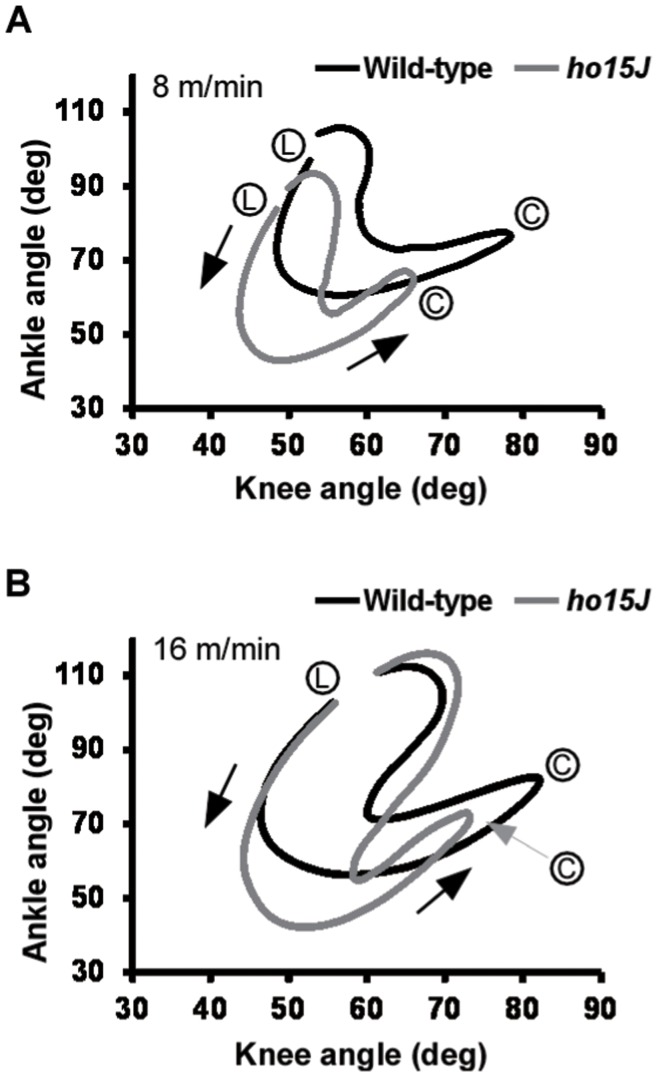
Mean interjoint coordination patterns during the step cycle. Each line illustrates the averaged data for interjoint coordination patterns of the ankle and knee joints in wild-type (black) and *ho15J* mice (gray). (L), foot lift at the beginning of the swing phase; (C), foot contact at the beginning of the stance phase. Arrows indicate the direction of angular motions. A: 8 m/min, B: 16 m/min.

### Electron Microscopy

For electron microscopic analysis, mice under deep pentobarbital anesthesia were perfused transcardially with 2% paraformaldehyde/2% glutaraldehyde in 0.1 M sodium phosphate buffer (PB, pH 7.2). Parasagittal microslicer sections of the cerebellum (300 µm) were postfixed for 2 h with 1% OsO_4_ in 0.1 M PB. After block staining in 1% aqueous uranyl acetate solution and dehydration with graded alcohols, sections were embedded in Epon 812. Ultrathin sections (70 nm) were made using an ultramicrotome (Leica, Nusslock, Germany), and stained with 2% uranyl acetate for 5 min and mixed lead solution for 2 min. Electron micrographs were taken from the molecular layer with an JEM-1230 electron microscope (JEOL, Tokyo, Japan) at x6000. The numbers of synapses between PFs and PC spines were counted using 10 micrographs taken randomly from cerebellum of each genotype. PF synapses in which the lengths of postsynaptic density and the active zone do not match are defined as “mismatched” synapses. PC spines that do not accompany PF terminals are defined as “free” spines.

**Figure 4:**
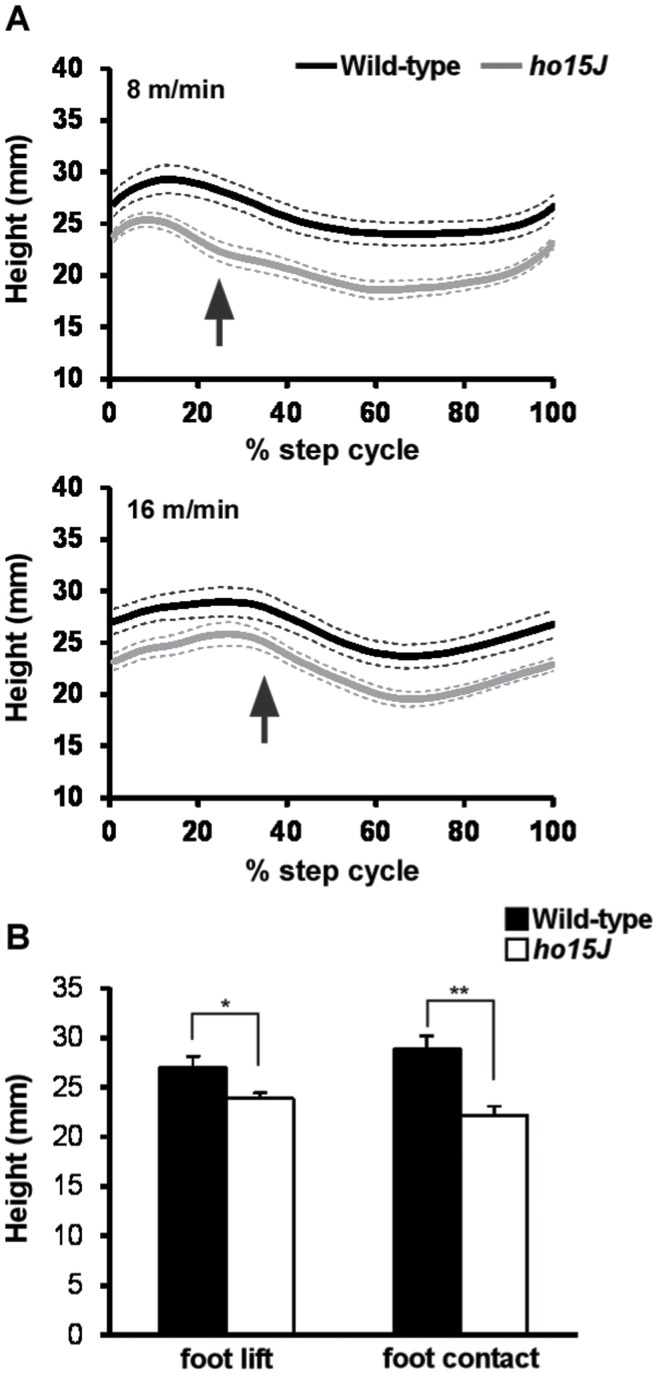
Great trochanter height during the step cycle. A: Average great trochanter height displacements during the step cycle at different treadmill speeds. Great trochanter heights during the step cycle were normalized. Arrows indicate the phase change point (swing to stance). Dotted lines indicate SEM. B: Great trochanter heights at foot lift and foot contact at 8 m/min. Error bars represent SEM. *, *p*<0.05; **, *p*<0.01.

### Statistical Analysis

Data are expressed as means ± standard error of the mean (SEM). Statistical comparisons between *ho15J* and wild-type mice were performed using Student’s *t*-tests. In all cases, *p*<0.05 was considered as statistically significant.

**Figure 5:**
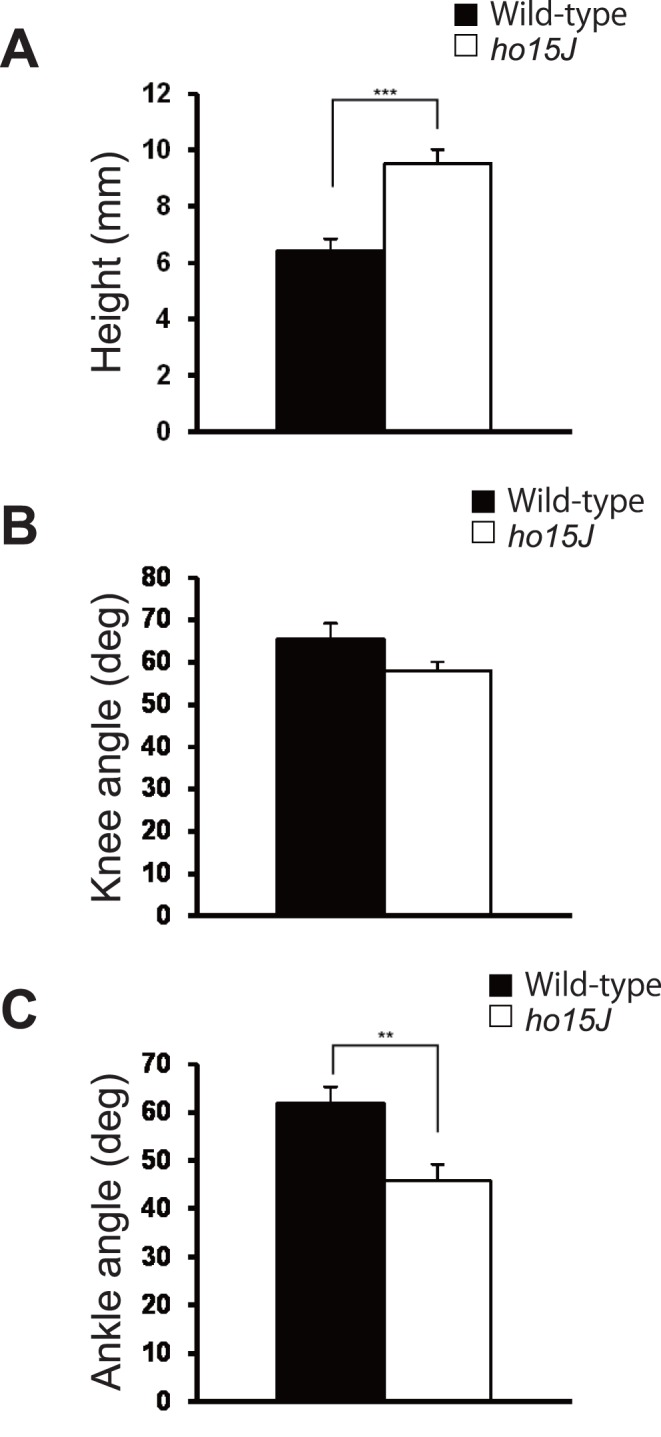
Kinematic analysis of maximal toe heights. A: Maximal toe heights at 8 m/min. The bar graph indicates the toe height at the highest point during the swing phase. B, C: Joint angles at the maximal toe heights at 8 m/min. Error bars represent SEM. **, *p*<0.01; ***, *p*<0.001.

**Figure 6:**
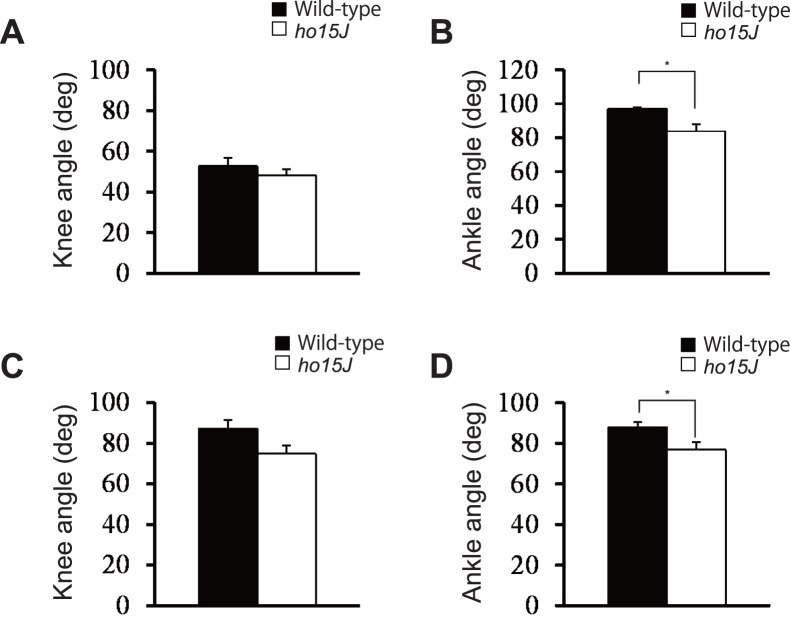
Joint angles at the start of the swing and stance phases. Average knee and ankle angles at foot lift and foot contact at 8 m/min. A, B: Foot lift. C, D: Foot contact. Error bars represent SEM. *, p<0.05.

## Results

Wild-type mice were able to walk on the treadmill at all speeds, but half of the *ho15J* mice were unable to walk stably and continuously on the treadmill when the belt speed was set at 24 m/min: the *ho15J* mice frequently fell off the treadmill. The *ho15J* mice that were able to walk on the treadmill at 24 m/min showed excessive extension of their hindlimbs and were conveyed backward (Data not shown). As half of the *ho15J* mice were unable to walk within our defined a step cycle at 24 m/min, we were not able to compare the *ho15J* mice to wild-type mice. Therefore, we restricted the following analyses to belt speeds of 8 and 16 m/min. In this study, we used averages of pooled data for wild-type and *ho15J* mice unless otherwise stated.

**Figure 7:**
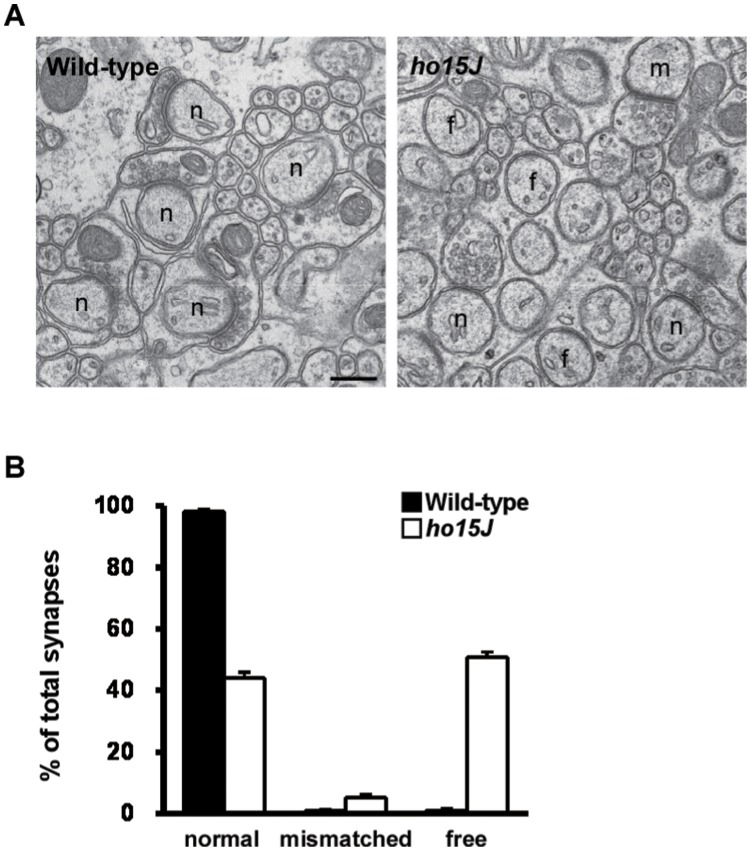
Electron microscopic analysis of parallel fiber–Purkinje cell synapses in wild-type and *ho15J* mice. A: Representative electron micrographs of the molecular layer of wild-type and *ho15J* cerebella at 3 months of age. In the wild-type cerebellum, all Purkinje cell spines contact parallel fiber terminals (n), whereas in the *ho15J* cerebellum, many free spines (f) and a few mismatched spines (m) are observed. Scale bar, 300 nm. B: Percentage of normal, mismatched, and free spines in the molecular layer of wild-type and *ho15J* cerebella. n = 10 micrographs per genotype.

### Temporal Parameters

The average durations of the step cycle, swing phase, and stance phase at 8 and 16 m/min are shown in Tables 1 and 2. At each speed, there were no significant differences between the wild-type and *ho15J* mice. Typical examples of the trajectories of the right hindlimbs of a mouse are illustrated in Figure 1. Although there were no significant differences in the temporal parameters of *ho15J* and control mice, the mutant mice did showed different hindlimb trajectories during treadmill locomotion compared to wild-type mice (see Fig. 1B). We therefore analyzed this peculiarity in more detail using the kinematic data.

### Spatial Parameters

Knee and ankle angular displacements during step cycles at different walking speeds are shown in Figure 2. Comparison of knee angular displacements during walking at 8 m/min showed that the knees of *ho15J* mice tended to be more flexed than those of wild-type mice at the phase shift point (the time of foot contact with the treadmill belt) (Figs. 2A, B). A similar difference was evident in the comparative ankle movements of the mutant and wild-type mice at the middle of the swing phase (Figs. 2C, D). However, at 16 m/min, the ankle angle of *ho15J* mice was greater than that of the wild-type mice in the last part of the stance phase.

The angles of excursion of the ankle relative to the knee are shown in Figure 3; these angles indicate interjoint coordination patterns of the ankle and knee joints. Each line shows the simultaneous angle motion at both joints across a step cycle. In the diagram, foot lift (L) is at the upper left and foot contact (C) is at the upper right. In wild-type mice, knee/ankle movement describes a crescent shape, similar to that reported previously in the normal rat [18]. In comparison with wild-type mice, knee/ankle movement in the *ho15J* mice showed displacement of the curve with clearly distinguishable differences in contour pattern (Fig. 3). This difference reflects the increased flexion of the ankle joint and the reduced range of motion of the knee joint. Thus, the analysis of *ho15J* mice indicates that the overall coordination between the knee and ankle joints was changed. Similar patterns were observed for the mice on the treadmill belt at 16 m/min. These findings suggest that *ho15J* mice have an abnormal pattern of coordination of the knee and ankle joints during treadmill locomotion.

In *ho15J* mice, great trochanter height was lower than in wild-type mice throughout the step cycle, regardless of the speed of walking (Fig. 4A). Great trochanter heights were significantly lower in *ho15J* mice than wild-type mice at the times of foot lift and foot contact with the treadmill belt at 8 m/min (foot lift: wild-type, 26.9±1.2 mm, *ho15J*, 23.9±0.6 mm, *t*(14) = 2.325, *p = *0.036; foot contact: wild-type, 28.9±1.3 mm, *ho15J*, 22.2±0.9 mm, *t*(14) = 4.136, *p = *0.001; Fig. 4B). At 16 m/min, great trochanter heights were also significantly lower in *ho15J* mice than in wild-type mice (foot lift: wild-type, 27.1±1.2 mm, *ho15J*, 23.2±0.8 mm, *t*(14) = 2.654, *p = *0.019; foot contact: wild-type, 28.7±1.3 mm, *ho15J*, 24.9±0.9 mm, *t*(14) = 2.445, *p = *0.028).

The *ho15J* mice exhibited apparently excessive toe elevation during the swing phase (Fig. 1B), therefore we analyzed maximal toe height and found that there was a significant difference in maximal toe height elevations between the mutant and wild-type mice at both 8 m/min (wild-type, 6.4±0.4 mm, *ho15J*, 9.5±0.5 mm, *t*(14) = 4.816, *p*<0.001; Fig. 5A) and 16 m/min (wild-type, 6.4±0.5 mm, *ho15J*, 8.6±0.8 mm, *t*(14) = 2.272, *p = *0.039, Data not shown). Taking into account angular displacements, it was possible that the excessive toe elevation was caused by hyperflexion of the ankle. To determine if this was the case, we examined angular displacements at the moment of maximal toe height (Figs. 5B, C). At 8 m/min, the knee angles of the mutant and wild-type mice were not significantly different (wild-type, 65.5±3.8 deg, *ho15J*, 58.0±2.1 deg, *t*(14) = 1.726, *p = *0.106). However, the ankles of *ho15J* mice were significantly more flexed than those of wild-type mice (wild-type, 62.0±3.3 deg, *ho15J*, 45.9±3.3 deg, *t*(14) = 3.453, *p = *0.004). Similar results were obtained from the analyses at 16 m/min (knee: wild-type, 63.7±4.0 deg, *ho15J,* 61.3±2.6 deg, *t*(14) = 0.486, *p = *0.635; ankle: wild-type, 66.0±4.9 deg, *ho15J*, 48.7±5.2 deg, *t*(14) = 2.413, *p = *0.030, Data not shown). To compare the differences in angular displacement between the mutant and wild-type mice further, we examined knee and ankle angles at the time of foot lift and of foot contact at 8 m/min (Fig. 6). No significant differences were found for knee angle (foot lift: wild-type, 52.7±4.6 deg, *ho15J*, 48.2±2.9 deg, *t*(14) = 0.830, *p = *0.421; foot contact: wild-type, 87.2±4.2 deg, *ho15J*, 74.8±4.1 deg, *t*(14) = 2.104, *p = *0.054; Figs. 6A,C). By contrast, significant differences were identified for ankle angle (foot lift: wild-type, 97.0±3.0 deg, *ho15J*, 83.7±4.1 deg, *t*(14) = 2.597, *p = *0.021; foot contact: wild-type, 87.8±2.5 deg, *ho15J*, 76.7±3.8 deg, *t*(14) = 2.447, *p = *0.028; Fig. 6B, D).

### Morphometry of the PF-PC Synapses

During locomotion, the cerebellum receives signals on the activities of the spinal neuronal circuits that control limb movements through the spinocerebellar pathways. These signals are transmitted to PCs via the mossy fibers-granule cells-PFs. Although electron microscopic analyses have been previously reported for GluD2-knockout mice (C57BL/6 background), the morphology of PF-PC synapses in *ho15J* mice (C3H background) has not been analyzed in detail [11]. Since genetic backgrounds of mice could play non-negligible effects on the cerebellar phenotypes [24], we performed an electron microscopic analysis of the ultrastructure of the cerebellum to compare the number and organization of PF-PC synapses in wild-type and *ho15J* mice (Fig. 7). In the wild-type cerebellum, all PC spines contacted with PF terminals. However, in the *ho15J* mouse cerebellum, only about 40% of spines contacted PF terminals; the remainder were mainly present as free spines, a few were mismatched spines. These findings indicate that the number of PF-PC synapses is markedly reduced in *ho15J* mice.

## Discussion

In the present study, gait ataxia in *ho15J* mice was studied by kinematic analysis of hindlimb movements during treadmill locomotion. The cerebellum of the *ho15J* mice is characterized by a 40% reduction in the number of PF-PC synapses compared with wild-type mice. There were no difference in general temporal parameters of treadmill locomotion between the *ho15J* and wild-type mice. However, the mutant and wild-type mice did show two other clear differences: first, the *ho15J* mice showed excessive toe elevation during the swing phase compared to wild-type mice; second, the *ho15J* mice showed hyperflexion of ankle joints. The angular displacement in the ankles of *ho15J* mice was larger than that of the wild-type mice at the moment of maximal toe height, and around the time of foot contact and lift. These results suggest that excessive toe elevation and severe hyperflexion of the ankle during the swing phase characterize gait ataxia in *ho15J* mice. Similarly exaggerated toe elevations during the swing phase have been also observed in *lurcher* mice, in which PCs are totally absent [15], and in mice with other *hotfoot* mutations [20].

### Gait Ataxia in Cerebellar Mutant Mice

Excessive toe elevation may be a characteristic feature of locomotion in quadrupedal animals with cerebellar dysfunction. Fortier et al. [15] reported that electromyographic (EMG) activities of the tibialis anterior (ankle flexors) in *lurcher* mice continue longer (nearly twice as long) as those of the wild-type mice, whereas those of the triceps surae (ankle extensors) in *lurcher* mice are characterized with weakened tonic activity between irregular small bursts. The prolonged activation of the ankle flexors might be responsible for the excessive toe elevation during the swing phase, and weakened activation in the ankle extensors might induce attenuated tension of the antigravity muscles in *ho15J* mice. In the present study, although we do not have EMG data for the *ho15J* mice, it is possible that prolonged activity of the ankle flexors may be responsible for hyperflexion of the ankle. Furthermore, the lower heights of the great trochanter might be induced by the weakened activities of the antigravity muscles which included the ankle extensors.

A previous study on *lurcher* mice reported that latencies before falling from the belt during treadmill locomotion are shorter at relatively low treadmill speeds (3 and 6 m/min) compared to wild-type controls [19]. In contrast, *ho15J* mice can walk at higher treadmill speeds: in this study, we were able to obtain data at 16 m/min (and up to 24 m/min for a few of the mice). The PCs of *lurcher* mice degenerate during early postnatal life [15] and the animals also have severe degeneration of their cerebellar granule cells and inferior olivary neurons [21], [22], [23]. In comparison, mice with a GluD2 mutation, such as *hotfoot*, GluD2 nulls or *ho15J* mice, show a marked decrease in the number of PF-PC synapses regardless of the presence of PCs ([6], [9], [24], present study). The mice fell off the treadmill at a speed of 24 m/min although they could walk less than 16 m/min, indicating that the damage in cerebellum result in the difficulty in adapting more serious conditions.

### Cerebellar Locomotor Control

During locomotion, the vermis and intermediate region of the cerebellum receive information originating from the spinal stepping generator or peripheral somatosensory receptors through the spinocerebellar pathways. This information is conveyed to PCs via the mossy fiber-granule cell-PF afferents. PCs in turn issue output signals to modulate activity of the brainstem descending tract neurons involved in locomotion. In this way, the cerebellum and spinal cord form the spinocerebellar loop. Afferent signals related to limb movements reach the PCs via the dorsal spinocerebellar tract (DSCT), the ventral spinocerebellar tract (VSCT), and the spinoreticulocerebellar pathway (SRCP) [25], [26]. In particular, the DSCT mediates global parameters of hindlimb kinematics and is considered to be important for appropriate limb movement control during locomotion [27], [28], [29]. In cats, reversible inactivation of the cerebellar intermediate cortex at lobules IV and V by cooling induces hyperflexion of the ipsilateral hindlimb and forelimb during treadmill locomotion [30], [31], [32]. As the number of PF-PC synapses is considerably reduced in *ho15J* mice, dysfunction of the PF-PC synapses might underlie the impairment of hindlimb movements during locomotion. Future studies to record the neuronal firing activities in the cerebellum during abnormal gait in these mutant mice will help to elucidate the neuronal mechanisms underlying gait control.

### Human Gait Ataxia

Human gait ataxia as a result of cerebellar damage or disease is characterized by impaired interjoint coordination between the ankle and knee. Patients with cerebellar degeneration show a reduced stride length when walking on a floor at a self-determined pace [33], a reduction in the range of angular motions of the limb joints when walking quickly on a level surface [3], and increased temporal variability, particularly during the swing phase [34]. Abnormalities in interjoint coordination have also been observed in patients walking on a variably inclined surface [35]. These observations suggest that the cerebellum has a crucial role for adjusting the coordination pattern among the different joints, especially in adaptability to externally imposed constraints. The gait ataxia of *ho15J* mice was also characterized by impaired interjoint coordination between the ankle and knee, which was attributed to the increased flexion of the ankle joint and the reduced range of motion of the knee joint. Human patients with cerebellar damage or disease may choose a strategy involving decreased motion of the joints in order to avoid falling.

Despite the obvious differences between quadrupedal and bipedal locomotion, ataxic mutant mice, such as *ho15J* mice, are capable of providing insights into human cerebellar gait ataxia. It is likely that dysfunctions in the cerebellar neuronal circuits result in a reduction in the level of muscle tone of the hindlimb muscles, which, in turn, causes severe hyperflexion of the ankle joints and lower great trochanter height in *ho15J* mice; this outcome is analogous to the ataxic gait of cerebellar patients.
